# An internet-based emotion regulation intervention versus no intervention for nonsuicidal self-injury in adolescents: study protocol for a feasibility trial

**DOI:** 10.1186/s40814-021-00785-4

**Published:** 2021-02-06

**Authors:** Britt Morthorst, Lotte Rubæk, Jane Lindschou, Janus Christian Jakobsen, Christian Gluud, Johan Bjureberg, Clara Hellner, Bo Møhl, Anne Katrine Pagsberg

**Affiliations:** 1 Research Unit, Child and Adolescent Mental Health Services, The Capital Region of Denmark, Gentofte Hospitalsvej 15, 2900 Hellerup, Denmark; 2 Team of Self-Injury, Child and Adolescent Mental Health Services, The Capital Region of Denmark, Lersøpark allé 107, 2100 Copenhagen Ø, Denmark; 3 Copenhagen Trial Unit, Centre for Clinical Intervention Research, Rigshospitalet, Blegdamsvej 9, 2100 Copenhagen Ø, Denmark; 4 Department of Regional Health Research, The Faculty of Health Sciences, University of Southern Denmark, Odense, Denmark; 5 Centre for Psychiatry Research, Department of Clinical Neuroscience, Karolinska Institutet, & Stockholm Health Care Services, Stockholm County Council, Stockholms läns sjukvårdsområde (SLSO), Sachsgatan 10, 118 61 Stockholm, Sweden; 6 Department of Communication and Psychology, Aalborg University, Teglgårds Plads 1 (Nordkraft), 9200 Aalborg, Denmark

**Keywords:** Non-suicidal self-injury, Emotion regulation individual therapy for adolescents (ERITA), Internet-based intervention, Randomised feasibility trial

## Abstract

**Background:**

Non-suicidal self-injury (NSSI) has gained increased attention in recent years due to increased prevalence, especially among adolescents. Evidence-based interventions for NSSI are sparse. Emotion regulation individual therapy for adolescents (ERITA) is an online intervention that needs investigation. Non-randomised studies suggest ERITA improves emotion regulations skills and reduces NSSI frequency. Before conducting a large pragmatic randomised clinical trial, we aim to investigate the feasibility of ERITA in Denmark.

**Methods:**

A randomised, parallel group feasibility trial comparing ERITA as add on to treatment as usual versus treatment as usual in 30 adolescents age 13–17 years with recurrent NSSI referred to outpatient clinics in The Child and Adolescent Mental Health Services in the Capital Region of Denmark. Feasibility outcomes are (1) completion of follow-up, (2) the fraction of eligible participants who consent to inclusion and randomisation and (3) compliance with the intervention. Clinical outcomes such as self-injury frequency and the ability to regulate emotions will be investigated exploratorily.

**Discussion:**

Internet-based interventions are assumed to be appealing to adolescents by being easily accessible and easy to navigate by tech natives. Disclosure of self-injury behaviour may be facilitated by an online intervention. The evidence for self-injury specific treatment needs to be extended but prior to a large clinical trial, the feasibility of methods and procedures must be assessed.

**Trial registration:**

ClinicalTrials.Gov Identifier: NCT04243603.

## Background

Non-suicidal self-injury (NSSI) has gained increased research attention in recent years since it is a widespread phenomenon across vulnerable groups often with severely damaging behaviours with or without further distinct psychopathology, especially among adolescents. NSSI prevalence and incidence are difficult to estimate due to highly heterogeneous studies, particularly in non-clinical samples [1]; however, an overall prevalence of 17% among adolescents has been found in recent meta-analyses [2, 3]. Prevalence in community surveys are associated with great underreporting [2], and hidden numbers are referred to as the ‘bottom of the iceberg’ [4]. A prevalence of 33% has been observed in a community sample of 15–17-year-old Scandinavian teenagers having self-injured at least once the last year, whist 41% of these did so repeatedly (> 11 episodes the last year) [5]. A lifetime prevalence of 15% has been shown in US college students following a web-based wellbeing survey [6]. Likewise, incidence rates of NSSI are not easily estimated. A systematic review investigating the longitudinal course of NSSI and deliberate self-harm more broadly, included 32 cohort studies (69% on NSSI exclusively (*n* = 969,197), 75% in community samples) and one of the main findings was that NSSI is a fluctuating behaviour difficult to assess [7]. A neutral NSSI course was suggested, however, with an observed increase in young adolescence and a decrease in late adolescence or young adulthood [7]. Studies investigating psychiatric populations of youth have shown estimates between 50 and 75% of patients engaging in self-injurious behaviour, especially in youth with emotional dysregulation and instability in relationships [8, 9]. In general, psychosocial stressors like hopelessness [10], depressive symptoms [7], sexual dysphoria [11], peer victimization [12], and family dysfunctional aspects are risk factors associated with NSSI [13]. Gender differences exist within the NSSI phenomenon, with more girls injuring themselves than boys [6, 7, 14]. NSSI is a risk factor for adverse outcomes in young people [15, 16], among them suicidal behaviour [17–19] and suicide [20, 21]. Since this behaviour is mainly observed during adolescence, researchers have hypothesized that trauma and childhood maltreatment are associated with increased risk for NSSI [22–24]. Perceived low social family and peer support and lack of persons from which to seek advice are factors reported by youth engaging in repeated NSSI [25, 26].

Why do so many young people self-injure? NSSI functions are described in the model of Nock and Prinstein as a distinction between intrapersonal (i.e. reinforced by oneself; e.g. emotion regulation) and interpersonal properties (i.e. reinforced by others; e.g. attention or communication) [27]. Most young people engaging in NSSI state intrapersonal functions to be the most prominent function, whilst interpersonal functions are less frequently stated [28]. Difficulty in emotion regulation has been investigated in a meta-analysis, which found that more sever emotion dysregulation was associated with higher risk of NSSI [29]. An anonymous, American student survey (*n* = 1243; mean age 21.5, SD = 4.2 years) found that 15% engaged in NSSI and 43% of those stated ‘coping with uncomfortable feelings’ as the reason for initiating this behaviour [26]. Compared to social motives, the survey participants endorsed emotion regulation as the most common reason for NSSI and repeating this.

The mentioned extensive hidden numbers may be related to the aspects of disclosing self-injurious behaviour to peers, parents, or health care providers [30]. The student survey found that 59% of their study population had disclosed their self-injurious behaviour to relatives or health professionals with the experience that this was not helpful [26]. Lack of knowledge and the inability to cope with children’s negative emotions are associated with the perceived unhelpfulness of NSSI disclosure [26]. A need for social support may direct NSSI engaging youth towards peers. However, young people greatly influence each other, and it has been found that NSSI can be both initiated and maintained by social and environmental factors. This influence within groups has been observed in community samples [31] as well as inpatient populations [32, 33]. Therefore, it is important to intervene and to investigate up-stream and low-cost prevention strategies [34] preferably without elements of group therapy. Evidence-based interventions for NSSI specifically are sparse [35]. Evidence may support cognitive behavioural therapy (CBT) for adult individuals engaging in repetitive NSSI; however, the randomised clinical trials are with high uncertainty and the effects only moderate [36]. Internet-based solutions are assumed to be appealing to young people with maladaptive behaviours due to experiences of stigmatization [30]. A systematic review and meta-analysis investigating the effect of internet-delivered cognitive behavioural therapy (ICBT) for children and adolescents with a variety of psychiatric (*k* = 11) as well as somatic (*k* = 14) conditions (24 studies, *N* = 1882) found moderate effect sizes in favour of ICBT compared with waitlist control in between group analysis (Hedge’s *g* = 0.62, 95% confidence interval (CI) 0.41 to 0.84) on a range of study-specific outcomes; however, study quality and ICBT content varied greatly across studies. Moreover, waitlist control may not be an adequate comparison condition to establish efficacy [37]. The pooled effect size for the ICBT interventions targeting the psychiatric conditions separately was (*k* = 11, *N* = 473; *g* = 1.27, 95% CI 0.96 to 1.59) [38] leaving online interventions to be further explored.

Emotion regulation individual therapy for adolescents (ERITA) has been developed to meet the need for short-term, effective and easily accessible treatment [39]. The therapeutic framework of ERITA encounters elements of emotion regulation group therapy (ERGT), cognitive behavioural therapy (CBT), dialectical behavioural therapy (DBT) and acceptance and commitment therapy (ACT) [40]. An uncontrolled feasibility study of individual face-to-face ERITA provided to adolescents engaging in NSSI (*N* = 17), which was conducted in Sweden suggested improved emotion regulations skills and significantly reduced past month NSSI frequency [40]. Further research from the same research group replicated their findings in a subsequent feasibility study investigating an internet-based version of ERITA (*N* = 25) [41]. In addition to the adolescent intervention, both feasibility studies included a parent intervention to increase the understanding of NSSI and to improve coping skills with the children’s negative emotions [41].

There is a need for replication of feasibility studies outside Sweden as well as randomised clinical trials testing specialised intervention for NSSI in this youth friendly format [38]. Before conducting larger pragmatic trials, we want to investigate the feasibility of ERITA in Denmark.

### Objective

The objective of this feasibility trial is to assess the feasibility of methods, procedures, and safety of internet-based ERITA in a Danish context. We compare ERITA as an add-on to treatment as usual (TAU) versus TAU alone in 13–17-year-old patients with NSSI referred to psychiatric services in the Capital Region of Denmark.

### Hypotheses

The primary hypotheses regarding feasibility are:

1. We expect ≥ 87% to complete follow-up questionnaires (data completion fraction at end of intervention).
2. We expect ≥ 40% of eligible patients and parents will be included by giving informed consent and proceed to randomisation.
3. We expect ≥ 73% of the participants will comply with experimental intervention and complete at least six modules out of 12 including an introduction.
4. We expect ≥ 73% of the participants’ parents will comply with experimental intervention and complete at least three out of six modules.

## Methods/Design

### Design

A randomised, parallel group, feasibility trial with blinded outcome assessment at end of the intervention.

### Study setting

Adolescents (*N* = 30; 13–17 years both inclusive) with recurrent NSSI referred to outpatient clinics in Child and Adolescent Mental Health Services (CAMHS) in the Capital Region of Denmark. We wish to include a representative sample of patients with a variety of diagnoses since all may present with comorbidity of NSSI.

### Inclusion criteria

- ≥ 5 non-suicidal self-injury episodes during the past year and ≥ 1 non-suicidal self-injury episodes during the past month assessed by the Deliberate Self-Harm Inventory, Youth version (DSHI-Y) [42].
- Age-appropriate Danish literacy assessed by referring clinicians and the self-injury team.
- At least one parent committing to participate in the parent program.
- Informed consent from parents/legal caretakers.
- Informed consent from the participant above 15 years of age.

### Exclusion criteria

- Elevated or imminent suicidal risk assessed by clinicians during routine screening procedure (that can be rated as no risk, elevated risk, or imminent risk) in need of admission or other life saving strategies.
- Lack of informed consent from parents/legal caretakers.
- Lack of informed consent from the participant above 15 years of age.

### Informed consent for trial participation

Detailed trial description and informed consent forms will be sent electronically to eligible participants and their parents linking personal ID numbers to secure mailbox. During the baseline interview after further assessment of eligibility, the clinicians will ask participants (adolescents > 15 years of age and both custody holders) to give informed consent by accessing this secure link and signing. Consents and assents will be stored directly in the research database, REDCap.

### Treatment as usual

Both the experimental group and the control group will receive TAU in CAMHS. TAU encounters a variety of clinical treatment and assessment offers, representing a highly inhomogeneous group of treatments, for instance: pharmacological treatment, family-based treatment, cognitive behavioural therapy, supportive counselling, and psychoeducation. Throughout the trial, the treatment responsibility is handled by clinicians providing TAU in CAMHS. In a subsequent large-scale trial, we expect TAU to be delivered equally in both arms and patients record data documented electronically, including treatment provision and duration will be obtained. Routine screening for suicidal behaviour is a part of the standard clinical assessment and treatment in CAMHS including safety screening and planning.

### ERITA

The ERITA intervention [40, 41] as add-on to TAU is a manualised internet-based therapy based on emotion regulation group therapy, cognitive behavioural therapy, dialectical behavioural therapy and acceptance and commitment therapy. The program consists of an introduction plus 11 modules ranging in content from psychoeducation, through emotional awareness training, and regulation of impulses and emotions by acceptance and validation (Table 1). The intervention also provides six modules for the parents focusing on NSSI and other risk-taking behaviours, emotional awareness, and validation skills (Table 1).

**Table 1:** An overview of the content of internet based ERITA

Adolescent intervention (module content)	Parent program (module content)
(1) Functions of non-suicidal self-injury	(1) Psychoeducation
(2) Impulse control	
(3) Functionality of emotions and emotional awareness	(2) Emotional awareness
(4) Primary vs. secondary emotions	
(5) Emotional avoidance / unwillingness vs. emotional acceptance / willingness	(3) Validation and invalidation
(6) Non-avoidant emotion regulation strategies	
(7) Implementing emotional approach and non-avoidant emotion regulation strategies	(4) Self-validation and self-invalidation
(8) Validation and emotional approach	
(9) Valued direction	(5) How to improve parenting in the long run / behavioral activation
(10) Repetition	
(11) Relapse prevention	(6) Summary and evaluation

ERITA is provided online and therapist-guided. The participants are expected to complete one module every week whilst the parents are expected to complete a module every second week. A mobile app is available to complement the online treatment. The app includes reminders of homework and skills and allows to report on both self-destructive behaviours and impulses daily. Weekly, electronically assessments of emotion regulation skills, NSSI, and potential risk behaviours will serve as intervention indicators for the therapist to guide the young ERITA participant during the intervention process. This intervention also includes a parent program with six modules focusing on psychoeducation and validations skills. The parents can review the adolescents’ chapters each week, but not the correspondence between the adolescents and the therapist. In contrary to previous evaluations of ERITA [40, 41], the parent part of ERITA is not therapist guided in the current trial.

### Strategy to improve adherence to intervention

If a participant fails to follow the treatment course by not accessing the web portal or by not replying to therapists’ messages, the intervention team will assertively reach out by phone to both the patient and the parents. It is expected that major parts of the dialogue with participants constitute motivation to adhere to the ERITA.

### Staff, qualification, and training

The staff in the self-injury team consists of psychologists and nurses, recruited with priority on experience within clinical child and adolescent psychiatry and with psychotherapy and special knowledge about NSSI. The staff is trained in the ERITA-manual [39] and will continually be supervised in the internet-based methods by clinicians and researchers of the Swedish National Self-Injury Project alongside Danish experts in the field. The self-injury team will warrant for the recruitment strategy by a continuous dialogue and update of staff in CAMHS.

### Outcomes

#### Feasibility outcomes are listed in order of importance

1. Completion of follow-up

Completion of follow-up will be defined as completing assessment of at least one clinical outcome (NSSI events) at end of intervention. If the number participants with completed outcomes is 26 out of 30, the fraction will be 87%, 95% confidence interval (CI) 75% to 99%. A follow-up fraction of 75% or more will be acceptable for a future trial whilst a fraction below 75% will impose serious problems of interpreting the trial result in a future large pragmatic trial.

2. The fraction of participants assessed for eligibility who consent to inclusion and randomisation

We will determine the fraction of potential participants as the number of eligible persons compared to the number of randomised persons. If the number of participants randomised out of the number of eligible persons is 30 out of 75, the fraction will be 40%, 95% CI 29% to 51%. A randomisation fraction of 29% or more will be acceptable for a future trial, whilst a fraction below 29% will impose serious problems of recruitment for a future large pragmatic trial.

3. Compliance—adolescents

Compliance with the experimental intervention will be defined as completing at least 6/11 ERITA sessions. The treatment platform will automatically register time for login and save the exercises that have been completed. If the number of compliant experimental participants is 11 out of 15, the fraction will be 73%, 95% CI 51% to 96%. A compliance fraction of 51% or more will be acceptable for a future trial whilst a fraction below 51% will impose serious problems of interpreting the trial result in a future large pragmatic trial.

4. Compliance—parents

Compliance with the experimental intervention will be defined as completing at least 3/6 ERITA sessions. The treatment platform will automatically register time for login and save the exercises that have been completed. If the number of compliant experimental participants is 11 out of 15, the fraction will be 73%, 95% CI 51% to 96%. A compliance fraction of 51% or more will be acceptable for a future trial whilst a fraction below 51% will impose serious problems of interpreting the trial result in a future large pragmatic trial.

### Clinical outcomes

We will assess participants at the baseline interview and at the end of the intervention at 12 weeks after randomisation. The clinical outcomes are planned for a future pragmatic randomised trial, and these will only be investigated in an exploratory manner in this feasibility trial.

#### Exploratory primary clinical outcome

- NSSI, assessed at end of intervention (12 weeks) by blinded outcome assessment by video conference or phone with DSHI-Y, continuous outcome [42].

#### Exploratory secondary clinical outcomes

- Quality of life at 12 weeks, assessed with Health-Related Quality of Life Questionnaire (Kidscreen-10) [43].
- Symptoms of depression, anxiety and stress at 12 weeks, assessed with Depression Anxiety Stress Scale (DASS-21) [44].
- Number of sick days the last month, assessed at 12 weeks.

#### Further exploratory clinical outcomes

- The proportion of participants with no NSSI during the last 4 weeks, assessed at 12 weeks follow-up (end of intervention).
- Difficulties in emotion regulation, assessed weekly during 12 weeks with Difficulties in Emotion Regulation Scale–16 Item Version (DERS-16) [45].
- Indirect self-destructive behaviours at 12 weeks, assessed with Borderline Symptom List (BSL-supplement) [46].
- Suicidal ideations, plans, and actions at 12 weeks, assessed with Columbia Suicide Rating Scale (C-SSRS) [47].
- Adolescent rated parents’ ability to cope with children's negative emotions at 6 weeks and 12 weeks, assessed with The Coping with Children’s Negative Emotions Scale (CCNES-APP) [48].
- Parent-rated perceived ability to cope with children's negative emotions at 6 weeks and 12 weeks, assessed with The Coping with Children's Negative Emotions Scale Adolescent (CCNES-A) [48].
- Adverse Events of therapy at 12 weeks assessed with Negative Effects Questionnaire (NEQ) [49].
- Strengths and difficulties assessed by Strengths and difficulties questionnaire (SDQ) assessed at end of intervention [50].
- Working alliance with online therapist at 4 and 8 weeks, assessed with Working Alliance Inventory, short version (WAI-SR) [51].

### Participant safety

#### TAU

Throughout the trial, the treatment responsibility is handled by clinicians providing TAU, including continuous risk assessment of suicidal behaviour as a part of routine clinical procedures and documentation. Patients receiving treatment in CAMHS and their families are instructed to attend the Child and Adolescent Mental Health Services emergency department in case of imminent suicidal risk. All families are carefully instructed on safety routines within the trial; hence, it is firmly stated in the participants’ instructions that therapists are only available during working hours. In case of imminent risk and no contact can be made, the intervention team will notify the clinical staff providing TAU and hereby ensure further acute clinical psychiatric assessment.

#### ERITA

As a part of the provision of ERITA, participants in the experimental arm will weekly fill out electronic questionnaires on emotion regulation and potential risk behaviour such as alcohol drinking or substance misuse as well as suicidal behaviour. The weekly assessments serve two purposes: firstly, to provide the ERITA therapists with data on NSSI and emotion regulation skills as a part of the provision of the intervention. Secondly, because internet-based interventions have not previously been applied in CAMHS, these weekly assessments ensure participant safety and facilitate detection of sudden deterioration of participants, including suicidal ideation. An individualised crisis plan will be established prior to treatment start, which will contain necessary contact information to acute health care services. If the patient during the research course is assessed to be at increased suicidal risk, a notification in the program will alert all team members and a therapist will contact both the patient and the parents next working day.

### Diagnostic assessments and outcome measures

#### Patient record data

**Fig. 1:**
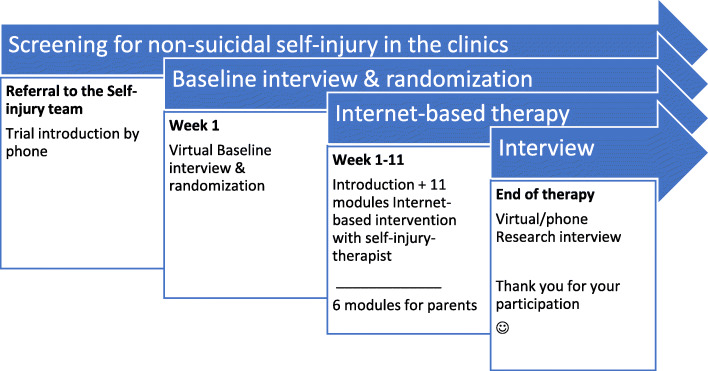
Participant timeline

Both the baseline and 12-week follow-up interviews are held virtually in secure meeting fora. Data obtained manually during the interviews are entered in an electronic research data base right after the interviews. None of the interviews are audio or video taped. The baseline interview is scheduled to take 2.5 h whilst the follow-up interview will take about 30 min. To minimise the baseline interview and assessment program, diagnosis codes will be obtained from patient records as a part of general clinical procedures. Also, data on the provision of TAU (treatment and length) for all participants will be obtained from the patients records by the end of treatment (12 weeks). Information on explorative outcomes and sick days the last 4 weeks will be obtained as self-reported at both baseline and follow-up by electronic questionnaires (Fig. 1).

#### Intervention data

During the internet-based intervention, data in the form of text as a part of the therapy will be provided; homework assignments will be presented, and therapists will write and interact with the participants, accordingly. The correspondence and interaction with participants and parents will differ in content according to individual needs but is expected to contain information on issues related to self-injury and coping strategies. Resource monitoring: we expect much of the therapeutic effort to focus on motivation to comply with modules and homework assignments. However, we do not yet know to what extent additional contact or support such as telephone calls or additional initiatives (e.g. emergency interventions) are required by the self-injury team.

#### Technical issues

A systematic monitoring of technical obstacles will be applied: login failures, forgotten passwords, inability to proceed during assignments or tasks, inability to hear audios or videos, and other unforeseen issues. All platform appearance will be monitored in detail including the need for web support. If technical issues appear or implementation as well as completion of the online intervention (misunderstanding of modules or assignments) then adjustments will be considered in dialogue with the developers to ensure the internal validity in future large-scale trials.

#### Resource allocation

A systematic monitoring of additional resources such as crisis phone calls, calls to treatment provider in TAU, parents’ consultations or rescheduling of baseline appointments will take place.

#### Sample size and power considerations

In this feasibility trial, we will include 30 participants corresponding to less than 10% of the probable sample size needed in a pragmatic large-scale trial. This corresponds to a power of 18% for the primary clinical outcome (self-injury episodes) in this trial, indicating that any positive result is purely exploratory and could be due to random error.

#### Analysis, randomisation, and blinding

The randomisation procedure will be provided by The Copenhagen Trial Unit (CTU) and be web-based. The allocation sequence will be computer-generated using block sizes of varying length concealed for the investigators. The allocation ratio is 1:1 and is blinded to the investigators.

Baseline characteristics will be assessed using descriptive statistics. The clinical exploratory outcomes will be analysed according to the principle of an intention-to-treat approach. In a subsequent large-scale trial, missing data will be accounted for by multiple imputations if data are missing at random. All data on participants will be analysed independently of adherence to treatment. We will analyse data using logistic regression for dichotomous outcomes and linear regression for continuous outcomes. Due to the nature of the intervention, a blinding of participants and therapists is not possible. Blinded outcome assessment will be performed. Statistical analyses will be performed by two blinded statisticians presenting independent reports. Based on the two blinded conclusions, two abstracts will be written and published (on a website).

## Discussion

### Design

The TEENS feasibility trial is the second in Scandinavia to investigate the feasibility of methods in a randomised design [52]. The acceptance and feasibility of the ERITA intervention has previously been investigated in Sweden in observational designs [40]. The feasibility outcomes in the TEENS trial are (1) the completion rate of at least one questionnaire at 12 weeks follow-up, the most important outcome; (2) the fraction of eligible patients randomised and included in the trial in order to implement a subsequent large-scale trial; and (3) the fraction of participants compliant with the intervention (six of eleven modules). We have estimated targets for the three feasibility outcomes and calculated corresponding confidence intervals. Each of the three targets can be argued to be either too optimistic or too pessimistic. Regarding the proportion of participants completing follow-up, it can be argued that any proportion less than 100% will be detrimental to any analysis. However, to accommodate the ‘real-life’ setting, we are also aware that it is not always possible to achieve a 100% follow-up. The assessment of the completion rate of at least one questionnaire at 12 weeks follow-up is essential in relation to the feasibility of a large-scale trial, where a high follow-up rate is important to potential effect sizes.

The TEENS feasibility trial will indicate if the recruitment strategy works. As a part of the implementation of the trial in CAMHS, a routine screening procedure for NSSI has been introduced to assess for inclusion criteria and eligibility. The efficacy of this screening procedure will be evaluated during the feasibility trial, as the number of referred patients followed by an assessment of eligibility and randomisation fraction.

The time frame of recruitment for the feasibility part is planned to 6 to 7 months (May–November 2020) which is assessed feasible. The randomisation procedure will also be tested, and the high-quality design will show if patients and their families want to participate in spite the risk of an allocation outcome not initially wished for. The feasibility part will also show if the parallel group design is accepted by the referring clinical staff. We expect the young patients to be tech-natives and with limited barriers to online activities and interventions. However, this feasibility trial will tell if the participants are motivated to comply with six out of eleven ERITA modules or if they are hard to engage during therapy.

### Internet-based treatment

No specific treatment targeting NSSI is provided in mental health services in either in- or out-patients settings in Denmark. The TEENS feasibility trial aims to fill this gap by testing the feasibility of a novel internet-based treatment among youth with psychiatric disorders. Provision of internet-based therapy in general is sparse in Denmark and for the time being only provided in one out of five regions. Here, online treatment for depression and anxiety has been offered adult patients since 2015 [53]. The internet-based treatments are therapist guided, self-help programs based on cognitive behavioural therapy; however, none of these programs are offered to adolescents. The TEENS feasibility trial will investigate if online treatment is appealing and easy to access for psychiatric youth and first and foremost if no serious adverse effects are observed. It has previously been observed that NSSI engaging youth have a better therapeutic response to online interventions compared to suicidal young people [54]; such therapeutic response, assessed as working alliance, will be evaluated during this trial. The potential to expand online interventions for other diagnoses within child and adolescent psychiatry may depend on the acceptance, feasibility and security of the online intervention in this trial; hence, this trial may inform future interventions.

### Parent involvement

This trial also includes a parent program with six modules focusing on psychoeducation and validations skills. Previous studies have found that an inability to cope with children’s negative emotions as well as invalidating manners are (risk) factors associated with NSSI [23, 25, 26], thus the maladaptive behaviours may be rooted in a family dysfunction. It is important to learn if the parent part is supportive for the youth which will be assessed by the Coping with Children’s Negative Emotions Scale for Adolescent rated by both the adolescent and the participating parent (CCNES-A, CCNES-APP) [48, 55].

### Strength and limitations

The ultimate strength of this trial is the randomised design. It is a strength that all the implementation of the intervention platform is managed by the intervention team; translation into Danish, layout and performance of audios and videos are matched to a Danish context. Platform operation and the therapeutic content is well known to trained staff members. Also, the trial organisation and implementation on an administrative level is a strength. A NSSI screening procedure implemented in CAMHS may contribute to the motivation of clinicians referring patients to the trial. We do not expect the internet-based intervention to have any serious adverse effects; however, this will be monitored closely by NEQ at post intervention assessment and by weekly assessments of self-harming behaviours including suicidal behaviour.

It is not possible to blind the participants nor the therapists which may impose a risk of bias despite blinded outcome assessment at end of intervention. The estimates of the feasibility outcome on completion is based on a rate of 88% as post treatment assessment observed in a previous Swedish feasibility study [41]; however, our estimates of the other feasibility benchmarks may impose a limitation due to lack of comparable investigations. We have estimated targets for the three feasibility outcomes and calculated corresponding confidence intervals. However, each of the three targets can be argued to be either too optimistic or too pessimistic. Regarding the proportion of participants completing follow-up, it can be argued that any proportion less than 100% will be detrimental to any analysis. The enrollment proportion and the compliance proportion are chosen from a pragmatic point of view, from what we estimate to be the lowest feasible numbers when conducting a large-scale randomised trial.

Further, the low sample size of 30 participants leaves any further explorative findings open to a large risk of random error. The weekly electronic online assessment of emotion regulation, NSSI and other potential risk behaviours in the experimental arm exclusively may impose a risk of confounding, since this is an opportunity of additional crisis intervention from the self-injury team in case of flag alerts. A potential limitation of the trial could be the provision of TAU; the lack of an experimental component in the TAU arm only, may lead to extended provision of TAU efforts in both frequency and duration causing an unsound effect which may overshadow or compromise the effect of the experimental intervention. Also, we will opt out to adjust from TAU efforts in the statistical analyses, because they will be provided post randomisation [56, 57].

Online interventions in psychiatric youth populations are pioneer work which implies an area of uncertainty; however, this high-quality feasibility trial with the recruitment and treatment responsibility held by mental health services secure both patient safety as well as outcome estimates in less risk of bias.

## Conclusions

The TEENS feasibility trial is important in several ways. Firstly, it provides feasibility assessment in a randomised design. Secondly, we investigate an intervention specifically developed to address NSSI by teaching and implementation of emotion regulations as well as validations skills in self-injuring youth and their parent. Thirdly, this is the first-time child and adolescent’s health care services provide internet-based therapy in Denmark; an opportunity to be further explored, potentially within other kinds of psychopathology such as for anxiety and depression. If the online intervention in the TEENS trial is accepted and completed among NSSI patients with no risk of side effects, then this may be extended to other groups of NSSI engaging youth, thus used as a potential up-stream prevention strategy.

## Trial status

Recruitment began mid-May 2020, and we expect the last participant to be included in November 2020.

## Data Availability

The trial investigators, selected members of the steering committee experts in the research database (BASS4) and investigators and statisticians at The Copenhagen Trial Unit will have access to the data. The datasets generated and analysed during this trial will not be made public available due to Danish law of data protection; however, a depersonalised final dataset will be sought to be made publicly available after publication of the trial results at The Danish National Archives (Rigsarkivet) as permitted by the Danish Data protection Agency.

## Abbreviations

NSSI: Non-suicidal self-injury
ERITA: Emotion regulation internet-based therapy for adolescents
CBT: Cognitive behavioural therapy
ICBT: Internet-delivered cognitive behavioural therapy
*g*: Hedge’s *g*
*n*: Number of included participants
ERGT: Emotion regulation group therapy
DBT: Dialectical behavioural therapy
ACT: Acceptance and commitment therapy
CAMHS: Child and Adolescent Mental Health Services
DSM-5: Diagnostic and Statistical Manual of Mental Disorders, Fifth Edition
REDCap: Research data base
CTU: The Copenhagen Trial Unit
Kidscreen-10: Health-Related Quality of Life Questionnaire
DASS-21: Depression Anxiety Stress Scale
DERS-16: Difficulties in Emotion Regulation Scale–16 item version
BSL-supplement: Borderline Symptom List
C-SSRS: Columbia Suicide Rating Scale
CCNES-APP: The Coping with Children’s Negative Emotions Scale
CCNES-A: The Coping with Children's Negative Emotions Scale Adolescent
NEQ: Negative Effects Questionnaire
SDQ: Strengths and Difficulties Questionnaire
WAI-SR: Working Alliance Inventory, short version
